# Where's the emergency? Improving emergency psychiatry experience for core trainees in Bath and North East Somerset (BaNES) and Gloucestershire Health and Care (GHC) localities

**DOI:** 10.1192/bjo.2021.422

**Published:** 2021-06-18

**Authors:** Tom Nutting, Sally Stuart, Francesca Hill

**Affiliations:** 1 BaNES; 2 GHC

## Abstract

**Aims:**

The Royal College of Psychiatry advises that core trainees should be involved in 50 first-line emergency assessments during their core training. This includes assessment of suicidal risk following self-harm at least monthly. Trainees in Bath and Gloucester are not meeting these requirements. We set up an emergency experience rota, with the aim of increasing trainees’ experience and confidence in assessment and management of emergency psychiatry.

**Method:**

An emergency experience rota was implemented in Bath in September 2017. Trainees were surveyed before and after their 6 month rotations. In cycle 1, trainees spent two weeks with the Crisis team and an additional three days with the Liaison team per rotation. In cycle 2, we made some modifications to the rota so that it was more flexible. This system was then adopted in Gloucester where trainees were encountering similar difficulties. We hope to complete cycle 3 across the two localities by July 2021.

**Result:**

From the initial two cycles conducted in Bath, post-change surveys showed an increase in trainees’ confidence in assessments in acute settings and completing risk assessments in cases of self-harm and suicidal ideation. All of the trainees who took part would recommend the experience to other trainees (100% (7/7)). In Gloucestershire, only pre-change data have been collected so far. A full analysis of all the results will be presented.

**Conclusion:**

The introduction of working time regulations such as the European Working Time Directive (2003) as well as local service reconfigurations leading to nurse-led liaison services and home treatment teams, have reduced the opportunity for trainees to undertake emergency assessments. Across the Severn Deanery, there is a discrepancy in the opportunity core trainees’ have to undertake emergency assessments – depending on their rota, stage of training, and services available. This difference in trainee experience, depending on locality, has been further impacted by COVID-19 and the introduction of cohorted wards.

Trainees in Bath and Gloucester are predominantly covering the wards during on-calls and, therefore, we set out to ensure that they are regularly rostered to obtain emergency experience, helping them meet their core training competences. Initial results from two cycles of an emergency / out-of-hours experience rota suggest increased experience and confidence in first-line emergency assessments, enabling them to work towards meeting their core training requirements.

